# Comparing student achievement in traditional learning with a combination of blended and flipped learning

**DOI:** 10.1002/nop2.492

**Published:** 2020-03-31

**Authors:** Suhaila Halasa, Nimer Abusalim, Mohammad Rayyan, Rose E. Constantino, Omayah Nassar, Huda Amre, Moayad Sharab, Insirah Qadri

**Affiliations:** ^1^ Maternal and Child Health Nursing Department School of Nursing The University of Jordan Amman Jordan; ^2^ School of Foreign Languages The University of Jordan Amman Jordan; ^3^ Department of Health and Community Systems School of Nursing University of Pittsburgh Pittsburgh PA USA; ^4^ Community Health Nursing Department School of Nursing The University of Jordan Amman Jordan

**Keywords:** academic achievement, blended learning, flipped classroom, nursing education, traditional learning

## Abstract

**Aim:**

The aim of this study was to investigate the effectiveness of blended learning with a flipped classroom design on student academic achievement in a Bachelor of Science in Nursing course.

**Design:**

A quasi‐experimental study.

**Method:**

Students were split into an experimental blended learning with a flipped classroom design group and a control group using the traditional, teacher‐centred learning method. Data were collected during spring 2018 (13.3 weeks) and student's grades for the registered course and their grade point average (GPA) were recorded.

**Results:**

Findings showed statistically significant increases in student grades in the experimental group. Predictability calculations also showed better achievement of learning outcomes if a blended learning with a flipped classroom design is continued to be used in the future.

## 1 INTRODUCTION

With the advancement of technology and the enrichments of online educational resources, educational institutions worldwide are witnessing a massive trend towards the integration of technology within their education and learning methodology (Harahap, Nasution, & Manurung, 2019). The centre of the learning process is being changed from teacher‐centred, direct instruction to student‐centred learning (Kintu, Zhu, & Kagambe, 2017). One way to achieve student‐centred learning is to make use of technology by incorporating interactive learning, video lessons and online interaction, where students can take the initiative in the learning process. Student‐centred learning can be achieved by either fully online courses or blended learning courses; the choice primarily depends on the institution's goals (Mestan, 2019; Suana, Distrik, Herlina, Maharta, & Putri, 2019; Vertejee, Somani, Allana, & Dias, 2015).

The University of Jordan has recently created a paradigm shift in the teaching–learning process from one based on teacher‐led instruction to one based on student‐centred learning, whereby the instructor's role is as an expert in the field who guides and facilitates students' active engagement in their own learning process. Towards this goal, the university has adopted a flipped learning/classroom approach combined with blended learning in a 2‐to‐1 ratio (2 hr per week in class and 1 hr per week at home) in several courses offered as a pilot study. In such a design, students learn the material they are assigned, at home, via video lessons, online interactions and/or web‐based material, before being presented with applications of what they learned during face‐to‐face class meetings. One such course is offered at the School of Nursing to second‐year nursing students. The focus of this study, therefore, is to test the effectiveness of blended learning (BL) combined with a flipped classroom (FC) approach in a course offered at the School of Nursing, with respect to student's academic achievement as measured through scores attained during course evaluation methods (examinations).

### 1.1 Background

#### 1.1.1 Blended learning

Blended learning is given several different names in the literature, for instance, mixed learning and hybrid learning (Barry & Abdullah Alhazmi, 2018). There is no set definition for blended learning in the literature (Hrastinski, 2019). Some define it broadly, with no focus on pedagogy changes involved, while others add details regarding the learning methods and pedagogies involved. For instance, Graham (2006, 2013) defines blended learning as a combination of traditional and online learning, while Finn and Bucceri (2004) suggest a combination of the best features of both traditional and online learning. On the other hand, Lotrecchiano, McDonald, Lyons, Long, and Zajicek‐Farber (2013) recognize a blend of structured and unstructured learning as blended learning.

Regarding location, some view blended learning as a learning approach that may occur solely in the classroom, but combining, within classroom time, various methodologies and encompassing various technological and online tools (Driscoll, 2002; Harvey, 2003). Additionally, Boelens, Van Laer, De Wever, and Elen (2015) suggest that blended learning is the combination of the best practices in traditional and online learning, meaning that some portion of class reduction may be involved. Cleary, some researchers consider blended learning to be part of in‐class activities with some distance online activities, while others view it as the integration of technology with traditional classroom education without splitting distance learning activities from classroom learning activities. Despite the body of literature on blended learning (Bliuc, Goodyear, & Ellis, 2007; Finn & Bucceri, 2004; Garrison & Kanuka, 2004; Garrison & Vaughan, 2008; Graham, 2006; Owston, York, & Murtha, 2013; Throne, 2003; Wilson & Smilanich, 2005; Yılmaz & Orhan, 2010; Zacharis, 2015), there is no consensus as to how to “blend” learning.

The advantages and disadvantages of online learning have been reported in the literature (Daniel, 2018; Dellana, Collins, & West, 2000; Hubackova & Semradova, 2016; Jones, Kukulska‐Hulme, & Mwanza‐Simwami, 2006; Kustijono& Zuhri, 2018; Mestan, 2019; Suana et al., 2019; Wang, 2010). Fully online or web‐based learning provides advantages such as flexibility, more student‐centred learning, a variety of learning pedagogies and self‐paced learning, but comes with disadvantages such as inhibiting face‐to‐face communication with other peers and requiring self‐regulated and self‐motivated students (Abrahamson, 1998; Browne, 2005).

Yılmaz and Orhan (2010) suggest that blending traditional face‐to‐face learning with online learning (a blended learning environment) can help solve the lack of interaction faced by learners in online education. However, the results in terms of student achievement regarding online learning vary. While some found positive results with the effectiveness of online learning on student achievement (Terry, Owens, & Macy, 2001), others found that both traditional learning and online learning created near similar results (Dellana et al., 2000; Iverson, Colky, & Cyboran, 2005).

#### 1.1.2 Blended learning and student learning outcomes

Several studies have investigated the effectiveness of blended learning (Akyüz & Samsa, 2009; Allen & Seaman, 2008; Haripersad & Naidoo, 2008; Hsu, 2011; Hsu & Hsieh, 2011a; Hsu & Hsieh, 2011b; Suana et al., 2019; Sugar, Martindale, & Crawley, 2007; Tynan, Ryan, & Lamont‐Mills, 2015; Vernadakis, Antoniou, Giannousi, Zetou, & Kioumourtzoglou, 2011; Woltering, Herrler, Spitzer, & Spreckelsen, 2009). “Effectiveness” generally means the student's academic achievement as measured by student grades in course assessments and examinations. Student grades are thus taken as a measure of the effectiveness of a certain learning model in achieving the desired learning outcomes.

Several studies find significant positive student performance for blended courses (Azizan, 2010; Bloemer& Swan, 2014; Harahap et al., 2019; Hughes, 2007; Jones & Chen, 2008; Lim & Morris, 2009; Woltering et al., 2009). Hughes (2007) increased tutor support and decreased class time and concluded that such a blended method, essentially, was proven beneficial to “at‐risk” students. A study conducted by Woltering et al. (2009) aimed at comparing problem‐based learning in both traditional learning and blended learning environments. They found that problem‐based learning in blended learning environments was significantly better achieved. Some studies report no statistically significant difference between traditional learning and blended learning with respect to achieved learning outcomes (Grandzol, 2004; Hsu & Hsieh 2011a; Hsu & Hsieh 2011b). Grandzol (2004) was interested in testing whether traditional and blended learning differed with regard to student achievement as evidenced by their grades in examinations. Grandzol, however, found no conclusive evidence that one method achieved more favourable results regarding learning outcomes. Hsu and Hsieh (2011a) and Hsu and Hsieh (2011b) study also showed no statistically significant difference between traditional and blended learning in terms of effectiveness towards learning outcomes achieved.

#### 1.1.3 Flipped learning

There is a growing body of research focusing on the flipped classroom (FC) design (Albert & Beatty, 2014; Kim, 2014; Kim, Khera, & Getman, 2014; O'Flaherty & Phillips, 2015), although the concept has been around for many years (Baepler, Walker, & Driessen, 2014; Bishop & Verleger, 2013; Gilboy, Heinerichs, & Pazzaglia, 2015). Several authors stress how flipped classrooms foster student engagement, resulting in better learning outcomes (Ash, 2012; Bergmann& Sams, 2012; Gilboy et al., 2015; Peterson, 2016; Pierce & Fox, 2012; Tune, Sturek, & Basile, 2013). In higher education, class time becomes time for further discussion of a topic that is already learned by students via pre‐recorded video lectures at home, or, in the case of health sciences, to take time to tackle more complex clinical case scenarios (Pluta, Richards, & Mutnick, 2013). We are left with testing the effectiveness of flipped learning designs on student achievement, given the University of Jordan's recent piloting of blended learning combined with flipped learning. However, as with blended learning, the literature on the effectiveness of flipped learning also shows conflicting results.

#### 1.1.4 Flipped learning and student learning outcomes

With respect to the flipped classroom model, research has produced conflicting results as to its effectiveness with respect to student achievement as measured by student grades. While some show improved measurable benefits to student grades (Johnson, 2013; Kong, 2014; Mason, Shuman, & Cook, 2013; Roach, 2014), others concluded no statistically significant difference (Blair, Maharaj, & Primus, 2016; Muzyk et al., 2015; Ryan & Reid, 2015).

With respect to the effectiveness and impact of the flipped classroom model on scores, Albert and Beatty (2014) compared traditional and flipped learning and found differences in student achievement as measured by student examination grades. The differences were not statistically significant for all examinations; the first and third examinations showed statistically significant differences in student scores favouring flipped learning, while the second examination showed no statistically significant difference. In the same way, Sahin, Cavlazoglu, and Zeytuncu (2015) showed that, in mathematics courses, students achieved significantly higher scores in the flipped section than in the traditional classroom.

However, not all research comparing traditional versus flipped classrooms in higher education turns out to be effective. In this sense, Muzyk et al. (2015) and Ryan and Reid (2015) provided results that do not show statistically significant differences in students' performance through examination scores in both formats and did not improve the students' performance at the end of the course.

Focusing attention more precisely on the health field, several higher education health‐related courses are, just as many institutions in general, seeking to enhance student‐centred learning (Melton, Chopak‐Foss, & Raychowdhury, 2008). Despite the popularity of flipped learning, research on flipped learning focusing on health sciences is scarce (Alemán, de Gea, & Mondéjar, 2011; Brettle& Raynor, 2013; Chen & Chuang, 2012; Dhir, Verma, Batta, & Mishra, 2017; Frith& Kee, 2003; Ruckert et al., 2014). According to Betihavas, Bridgman, Kornhaber, and Cross (2016), those studies regarding technology‐enhanced learning in nursing programmes yielded conflicting results: some neutral and some positive, and that none of the relevant studies evaluated the process of implementation of flipped classrooms combined with blended learning involving part at‐home and part in‐class tasks in a nursing setting, hence the significance of this study.

## 2 THE STUDY

### 2.1 Research questions

This study aimed to test the differences in the effectiveness of blended learning combined with flipped classrooms on the one hand, with traditional, face‐to‐face learning without a flipped classroom design. In particular, this research was concerned with the following research question.

RQ1: Are there statistically significant differences concerning achievement as assessed through student grades between the experimental group using blended learning with flipped learning and the control group using the traditional method without flipped learning?

This research is also concerned with how reliable a predictor; the results obtained for RQ1 are of future achievement of students if blended learning with flipped classrooms were to continue to be implemented. The following research question addresses this matter.

RQ2: To what extent can the results be predictable of academic achievement for the end GPA (graduation GPA), reflecting thus on the predictability of student achievement and effectiveness of each method. In other words, can the results of RQ1 be translated into future predictions regarding students' graduation GPA if BL + FC were implemented throughout the remainder of degree semesters?

### 2.2 Design

A quasi‐experimental method was used with an experimental and control group in the post‐test only. The first group was the control group, where the students studied the material through traditional learning. They were tested three times (first, second and final examination). The second group was the experimental group. Students studied the material via blended learning combined with a flipped classroom design and were tested with the same examinations (unified for control and experimental groups administered in large classrooms).

Accordingly, the design scheme of this study is:

Experimental Group (EG): *X* *O*; *N* = 59.
Control Group (CG): *O*; *N* = 66.

where “*O*” stands for academic achievement and “*X*” stands for processing using the blended learning method.

Details regarding the differences between the two groups are presented in Table 1.

**TABLE 1:** Details of differences in course activities and schedules between experimental and control groups

Course design particulars	Experimental group	Control groups
Learning Method	Blended + flipped learning	Traditional learning without flipped classrooms
Course Components and Schedule	Step 1: Students learn about that week's particular topic at home with the deduction of a third of actual class meetings (amounting to 2 actual face‐to‐face meetings out of 3). Step 2: Students meet face to face, in class for the remainder (2 out of 3 classes per week for 16 weeks)	New topics are offered in class only, during 3 one‐hour face‐to‐face lectures per week for 16 weeks
Activities	Students perform at‐home and in‐class activities, as follows: At‐home: Students prepare the material via watching a pre‐recording lecture online posted for them on the institution‐adopted learning management system, known as MOODLE. In‐class: Class begins with a student‐led discussion of key points; then, instructor offers more advanced questions and discussion given the additional time gained from not having to repeat nor teach the basics of that week's topic.	Students are first exposed to a certain week's topic from the instructor during 3 weekly face‐to‐face in‐class lectures. Students are given weekly assignments to do at home after being taught the material during class.
Assessment strategies	First, second and final examinations	First, second and final examinations

The control group met face to face in class three times a week (1 hr each), on Sundays, Tuesdays and Thursdays, and were given traditional teacher‐led lectures, with no online tasks. The control group was not asked to perform any extra coursework at home. They were only given the course syllabus, distributed via uploading to the Moodle platform (the university's LMS).

The experimental group met only twice a week (also 1 hr each), on Sundays and Tuesdays, and were given distance online tasks to complete the three‐credit‐hour course requirement (i.e. instead of Thursday's class). The course syllabus was uploaded for students on the Moodle, and all the topics, interactive strategies, videos and flipped classroom material were uploaded and available for students. Specifically, items concerning next week's topic were uploaded to the LMS on Tuesday nights with instructions for students to complete certain flipped classroom tasks, in preparation for the next class meeting (Sundays), where the topic they prepared at home would be discussed. This was to ensure that students prepared and attempted to learn the material on their own before arriving to the face‐to‐face meeting in class on Sundays, thus achieving a flipped classroom design and creating a student‐centred learning environment. Students were reminded via the LMS, to perform the tasks assigned to them and be ready for a class discussion on the topic during the first ten minutes of the class. Online tasks included watching an e‐lecture and reading material related to the upcoming topic.

## 3 METHODS

### 3.1 Participants

The participants of this study were second‐year nursing students who registered in the Foundation of Growth and Development course for the first time (4 sections), spring 2018, during the period of 28/1/2018–8/5/2018. All the students who registered in the 4 sections had agreed to participate in the study and were oriented about the purpose of the study before they registered in the course sections. Two of these sections were designed to apply the experimental method (blended learning with flipped classrooms), while the other two followed the traditional method of learning. Students had the choice to register in any section, thus achieving natural randomization; the result of which was 59 students in the experimental group and 66 in the control group.

### 3.2 The course invoked in the study

This study focused on a course offered for second‐year nursing students in the School of Nursing at the University of Jordan. The course title is “Foundation of Growth and Development” (Course Number: 0702102) and is one of the compulsory courses in the Bachelor of Science in Nursing programme with three credit hours per week. Students must take this course as a prerequisite to the “Children and Adolescent Health Nursing” course. The rationale for selecting this course is because students generally tend to have difficulties in achieving the course intended learning outcomes due to a lack of knowledge and understanding of the growth and development concepts and theories. The details of the course are offered in Table 2.

**TABLE 2:** Course details

Name of Course	Foundations of Growth and Development
Location	The University of Jordan—School of Nursing—Maternal & Child Health Dept.
Level	2nd‐year level—Core Course—Prerequisite to “Children and Adolescents Health Nursing Course”
Semester	Spring 2018
Total Number of Students	125
Course description	This course is designed to introduce students to the main concepts that are related to growth and development of humans throughout their life span. It focuses on the biological, psychosocial, cognitive, moral and spiritual characteristics of each developmental stage. The course introduces students to the strategies that can be used to help individuals of a particular developmental period attain optimal health. The framework of the course is based on the concept of health maintenance and promotion

### 3.3 Data collection instruments and procedures

Data were collected during the spring semester of 2018 (13.5 weeks), and students' grades (first, second and final examinations) for both groups were recorded. Before beginning the course, it was necessary to ensure that the two groups were equivalent in terms of previous student academic achievement levels (i.e. that, for instance, it was not the case that all of the high achievers were coincidently registered in one particular section). To verify the equivalence of the experimental and control group, the cumulative GPAs of the students were recorded at the beginning of the semester. As stated earlier, all students were second‐year students. Their GPAs included a cumulative average of their grades for previously taken courses throughout the preceding three semesters of their studies. Students' past cumulative GPAs were accessed by the university's intranet network available for faculty, which shows all student enrolments, their achieved grades and their overall cumulative GPAs at any given moment for all semesters completed combined. Students, as mentioned earlier, were briefed beforehand that this was going to be part of the participation in the experiment. Accordingly, for further confirmation of equivalency of control and experimental groups, a *t* test of the independent samples was conducted to verify the equivalence between the two groups according to the students' cumulative GPAs. Table 3 shows these results.

**TABLE 3:** Means, standard deviations and *t* test of independent samples of the cumulative average of students according to the experimental and control groups

Group	*N*	Mean	*SD*	*T*	*df*	Sig.
Experimental	59	2.77	0.566	1.684	123	.095
Control	66	2.60	0.535			

Abbreviations: *N*, Number of students; Sig., significant; *T*, *t* test.

Table 3 shows that the *t*‐value of the cumulative averages (entering overall GPAs) of students in the experimental and control groups was 1.684, which is not statistically significant. There are, therefore, no statistically significant differences concerning the entering cumulative averages between the experimental and control groups. This indicates that the two groups were equivalent in terms of previous academic achievement levels before applying the teaching method to the experimental group.

### 3.4 Data analysis

The data were analysed using the average, standard deviation and *t* test. The software used was SPSS Statistics.

### 3.5 Ethical considerations

This study was approved by the IRB Ethics Committee at the School of Nursing at the University of Jordan. The Foundation of Growth and Development course during spring 2018, was offered to students through 4 sections. At the beginning of the semester and before students registered the course, they were informed that two sections will be taught as blended learning and the other two by traditional methods. Students had the choice to register in any section they preferred. Furthermore, students were informed that all sections will cover the same course topics and have the same assignments and examinations to achieve the course intended learning outcomes. Students were informed that the researcher will review their grades and their previous cumulative grade point averages (GPAs) for the purpose of examining the effect of blended learning on their overall grades. Therefore, written consent explaining the purpose of the study was obtained from them.

## 4 RESULTS

### 4.1 Demographics

The demographic data showed that the total number of students in the 4 sections was 125 (99 females (79.2%) and 26 males (20.85), with an average age for males and females of 19 years old. All the students in the experimental group were Jordanian, while around 97% were Jordanian and 3% were Palestinian in the control group. The demographic characteristics of the participants (both groups) are shown in Table 4.

**TABLE 4:** Demographic characteristics of the participants (*N* = 125)

Group	*N*	Gender	Age	Nationality
Experimental	59	M: 13 (22.03%)	M: 19.6	59 (100%) Jordanian
		F: 46 (77.66%)	F: 19	
Control	66	M: 13 (19.70%)	M: 19.8	64 (96.96%) Jordanian
		F: 53 (80.30%)	F: 18.8	2 (3.03%) Palestine

### 4.2 Results related to the first research questions

RQ1: Are there statistically significant differences concerning achievement as assessed through student grades between the experimental group using blended with flipped learning and the control group using the traditional method without flipped learning?

To answer this question, the averages, standard deviations and *t* test for independent samples of students' academic achievement were calculated for both groups, as illustrated in Table 5. Students' total for all three examinations (first, second and final examination) was calculated, and the mean for each section was calculated. This is different from the method used by Albert and Beatty (2014), whereby achievement was taken separately for each examination (as discussed above). The intention for considering the total of all examinations was to avoid any external variables that may affect the data and avoid conflicting results (cf. Albert & Beatty, 2014). The mean for each group of total grades for these examinations (experimental and control) was also calculated. Table 5 details the results.

**TABLE 5:** The means, standard deviations and *t* test for independent samples of students' academic achievement between the experimental and control groups

Group	*N*	Mean	*SD*	*T*	*df*	Significance level
Experimental	59	77.77	7.985	2.968	123	.004*
Control	66	72.33	11.892			

Abbreviations: *N*, Number of students, *T*, *t* test, Sig., significant.

* Statistically significant at level .05.

Table 5 shows that the value of "*T*" of the achievement of students between the two groups according to the teaching method applied was 2.968, which is statistically significant. This indicates that there are statistically significant differences concerning the academic achievement of students between the experimental and control groups. The differences were in favour of the experimental group with a higher average than that of the control group. This means that the blended learning method improved the achievement of the experimental group. To calculate the effect of the teaching method, the following equation was used:

$$S_{C}=\sqrt[{2}]{\frac{\left(n_{1}-1\right)S_{1}^{2}+\left(n_{2}-1\right)S_{2}^{2}}{n_{1}+n_{2}-2}}$$

$$d=\frac{\left|{\bar{X}}_{1}-{\bar{X}}_{2}\right|}{S_{C}}$$

*d*: effect size.

Mean of the first sample.

Mean of the second sample.

Standard deviation for both samples.

*S_c_ = *((3698.09305 + 9192.27816)/123)^1/2^ = 10.23717568.

*d *= (77.77 − 72.33)/10.23717568 = 0.531.

The application of the effect size equation on the two independent groups using the *t* test showed that the value of the effect size was 0.531 and this is a medium effect size as indicated by Cohen (1988). This indicates that 53.1% of the differences concerning academic achievement between the experimental and control groups were due to the learning method using blended learning with a flipped classroom design.

### 4.3 Results related to the second question

RQ2: To what extent can the results offer a predictability of academic achievement for the end GPA (graduation GPA), reflecting thus on the predictability of student achievement and effectiveness of each method. In other words, can the results of RQ1 be translated into future predictions regarding students' graduation GPA if BL + FC were implemented throughout the remainder of degree semesters?

The aim of this question was to understand whether students' graduating GPA can be predicted to increase (as compared with traditional learning method) if the blended learning with flipped classroom design were applied throughout the remainder of the semesters of study (roughly 4 additional semesters). In other words, can it be predicted based on the results for RQ1 that students' graduating GPAs will increase if the experimental method (instead of the traditional learning method) were applied throughout the remainder of the semesters of study? To find the predictability of academic achievement, a linear regression analysis was conducted (Table 6).

**TABLE 6:** The predictability of academic achievement for the GPA

Model	Unstandardized Coefficients		*t*	Sig.	*R*	*R* Square	Adjusted *R* Square	*F*	Sig.
	B	*SE*							
(Constant)	0.014	0.263	0.053	.958	0.678	0.460	0.455	104.667	.000*
Achievement	0.036	0.003	10.231	.000*					

* Statistically significant at level .05.

Table 6 shows that the correlation coefficient between achievement and cumulative averages was 0.687, statistically significant at 0.05. This indicates that achievement affects and predicts the cumulative average of the students. The predictability of academic achievement for the GPA was 0.460, indicating that achievement accounts for 46% of the student's cumulative average. This is statistically significant at the level of significance. This indicates that 46% of the variation of the cumulative means accounts for students' achievement. Conversely, 46% of the variation in achievement is accounted for by students' cumulative average; 0.54% is accounted for by other variables. Accordingly, if blended learning with a flipped classroom design were to be implemented for the remainder of semesters until graduation, it is predicted that students' overall GPAs would increase (a separate study will need to be conducted to predict the extent of increase in detail).

## 5 DISCUSSION

Regarding the first question, the results revealed that students achieved higher grades in the blended learning group, and this is congruent with the study conducted by Harahap et al. (2019). The findings of a study conducted by Peterson (2016) were congruent with the results of this recent study where students had better academic achievements and were more satisfied with the overall statistics course in that study compared with students who had been taught the course through the traditional learning strategies. Nursing students who had been taught the clinical skills via blended learning received higher scores in relation to motivation and attitude towards learning professional skills (Coyne et al., 2018). Regarding the second question, the student's academic achievement in the course significantly predicts their overall GPA. Given such results of predictability coupled with the previous result of blended learning students achieving significantly higher grades than the non‐blended learning group, it can be said that blended learning is predicted to increase students' GPAs by the end of their university studies, if implemented throughout the remainder of their studies.

These findings concerning students achieving higher grades in blended learning classes are consistent with the findings of other related research (Azizan, 2010; Fortin, Viger, Deslandes, Callimaci, & Desforges, 2019; Hughes, 2007; Jones & Chen, 2008; Lim & Morris, 2009; Woltering et al., 2009; Yu & Du, 2019). Furthermore, an experimental study conducted by Sung, Kwon, and Ryu (2008) revealed that blended groups had a higher statistically significant level of knowledge and satisfaction related to administration of medication skills and knowledge compared with the control group, while there were no statistically significant differences between the groups in relation to self‐efficacy. Choi and Kim (2018) conducted a quasi‐experimental design to evaluate the effectiveness of an educational programme related to smoking cessation based on blended learning in improving nursing students' competence and motivation to conduct a smoking cessation intervention for smokers. The results showed that the educational programme adopting blended learning was significantly effective in developing nursing students' competences and motivation in conducting a successful programme.

None of the previously mentioned studies, however, entertained the idea of combining both blended learning and flipped classrooms. The statistically significant differences found between the two groups can be summarized as follows. First, students seem to understand the material better when having previously prepared it or for it at home (Gaughan, 2014; Roach, 2014). This, in turn, leads to the second point, namely that students in the experimental group are engaged to envision higher levels of Bloom's taxonomy given the extra time derived from freedom from face‐to‐face meetings; this is supported by the study conducted by Becker et al., (2017); Park, Woo, and Yoo (2016). Higher‐order thinking skills and more advanced activities are easily achieved in class when students have already prepared the basics of the topic at home (Gilboy et al., 2015; Johnson, 2013; Kong, 2014). The third point clearly noticed is the fact that blended learning offers student‐paced learning catering to the differences in learning styles and speed of each student, along with the added benefit of a flipped classroom which nudges students to become familiar with the material weekly, instead of the night before an examination, which tends to happen with traditional learning.

Given the results previously outlined, we suggest that the best teaching practices that create engaged students, taking the lead in their acquisition of knowledge, come from models of learning that are quite different from the traditional model. In educating nurses and healthcare providers, integrating blended learning within diverse learning methodologies enhances communication skills and improves self‐efficacy among nursing students (Shorey et al., 2018). As discussed earlier, blended learning alone does not ensure student‐centred learning; for instance, teaching a certain topic to students throughout the week in class only to give them the last day of the week off to perform homework on the same topic at home does not alone achieve student‐led learning, but, in fact, maintains the spoon‐feeding method, as evidenced by the literature citing negative results with blended learning mentioned above. Our research indicates that when students prepare the material at home via a flipped learning design, by watching a pre‐recorded lecture video, for instance and in some cases, being moved or inspired to search the Internet for more information on the topic before coming to class, we can then safely say that students are learning on their own and teachers only serve as guides or helping experts in the field.

### 5.1 Limitations

The main limitation of this study is the relatively small sample size (125 students in only 4 sections). Given that the blended learning and flipped classroom method was in the piloting phase at the University of Jordan, not many sections, nor blended learning strategy‐trained faculty members were available at the time. Another limitation is related to the students. In particular, students self‐selected which section they registered in; this may have had some influence on the study outcomes, even though the statistical analysis of previous GPAs shows no statistically significant difference between the two groups. A further limitation regarding students' self‐allocation to the sections was the fact that, since it was announced beforehand which sections were going to be taught as blended learning, it could be the case that the more technology‐comfortable students registered in the blended learning sections. If they were not given such information, then we would expect that each section would have some tech‐savvy students and some that are not comfortable with technology use. Could students have achieved the same results regardless of whether they were comfortable with technology use or not? Again, such a factor may have affected the results of this study.

### 5.2 Suggestions for future research

Future research may wish to replicate this study on a larger scale. Future studies could also aim to test, compare and contrast, in similar or dissimilar settings, the differences in student achievement between blended learning (with class time reduction) and flipped learning combined on the one hand, with flipped learning without reducing class time. Replicating this study in settings other than nursing schools is also recommended. In particular, researchers may wish to see whether similar results hold for other disciplines, such as medicine, pharmacy, social work, dentistry, rehabilitation science and other health disciplines. Moreover, researchers may focus on younger study participants to examine whether similar results could be obtained.

## 6 CONCLUSION

Despite the importance of using blended learning and online learning in general, there are many challenges facing educational programmes in the application and implementation of blended and online programmes in the educational curriculum. These challenges could be technical (the ability of the students and educators to use the technology successfully) and/or organizational (understanding, encouraging and facilitating of the manager for the implementation of blended learning). For the instructional design, when technology is adopted, more attention needs to be provided on how to teach, matching the best technology to achieve the indented learning outcomes, thus producing more well‐equipped nursing professionals. Professional nurses, educators and clinicians need to integrate and adopt blended learning as a learning strategy. Blended learning combined with flipped classrooms, as this study shows, allows nursing students to become self‐directed autonomous learners, thus enhancing nursing competencies effectively.

## CONFLICT OF INTEREST

The authors declare no conflict of interest to report.

## AUTHORS' CONTRIBUTIONS

Suhaila Halasa: Discussion, submission and corresponding author. NimerAbusalim: Results and analysis. Mohammad Rayyan: Results and analysis. Rose E. Constantino: Design, methods, data collection and review of the final draft. OmayahNassar: Discussion. Huda Amre: Introduction and background. MoayadSharab: Conclusion and background. InsirahQadri: Conclusion and review of the first draft.
